# A computational study of aortic insufficiency in patients supported with continuous flow left ventricular assist devices: Is it time for a paradigm shift in management?

**DOI:** 10.3389/fcvm.2022.933321

**Published:** 2022-10-20

**Authors:** Jonathan Grinstein, Pablo J. Blanco, Carlos A. Bulant, Ryo Torii, Christos V. Bourantas, Pedro A. Lemos, Hector M. Garcia-Garcia

**Affiliations:** ^1^Section of Cardiology, Department of Medicine, University of Chicago, Chicago, IL, United States; ^2^National Laboratory for Scientific Computing, Petrópolis, Brazil; ^3^National Scientific and Technical Research Council, Buenos Aires, Argentina; ^4^Department of Mechanical Engineering, University College of London, London, United Kingdom; ^5^Barts Heart Centre, London, United Kingdom; ^6^Heart Institute, University of São Paulo Medical School, São Paulo, Brazil; ^7^Hospital Israelita Albert Einstein, São Paulo, Brazil; ^8^MedStar Cardiovascular Research Network, Washington, DC, United States

**Keywords:** aortic insufficiency (AI), left ventricular assist device (LVAD), computational fluid dynamics, myocardial efficiency, right ventricular (RV) function

## Abstract

**Background:**

*De novo* aortic insufficiency (AI) following continuous flow left ventricular assist device (CF-LVAD) implantation is a common complication. Traditional early management utilizes speed augmentation to overcome the regurgitant flow in an attempt to augment net forward flow, but this strategy increases the aortic transvalvular gradient which predisposes the patient to progressive aortic valve pathology and may have deleterious effects on aortic shear stress and right ventricular (RV) function.

**Materials and methods:**

We employed a closed-loop lumped-parameter mathematical model of the cardiovascular system including the four cardiac chambers with corresponding valves, pulmonary and systemic circulations, and the LVAD. The model is used to generate boundary conditions which are prescribed in blood flow simulations performed in a three-dimensional (3D) model of the ascending aorta, aortic arch, and thoracic descending aorta. Using the models, impact of various patient management strategies, including speed augmentation and pharmacological treatment on systemic and pulmonary (PA) vasculature, were investigated for four typical phenotypes of LVAD patients with varying degrees of RV to PA coupling and AI severity.

**Results:**

The introduction of mild/moderate or severe AI to the coupled RV and pulmonary artery at a speed of 5,500 RPM led to a reduction in net flow from 5.4 L/min (no AI) to 4.5 L/min (mild/moderate) to 2.1 L/min (severe). RV coupling ratio (Ees/Ea) decreased from 1.01 (no AI) to 0.96 (mild/moderate) to 0.76 (severe). Increasing LVAD speed to 6,400 RPM in the severe AI and coupled scenario, led to a 42% increase in net flow and a 16% increase in regurgitant flow (RF) with a nominal decrease of 1.6% in RV myocardial oxygen consumption (MVO2). Blood pressure control with the coupled RV with severe AI at 5,500 RPM led to an 81% increase in net flow with a 15% reduction of RF and an 8% reduction in RV MVO2. With an uncoupled RV, the introduction of mild/moderate or severe AI at a speed of 5,500 RPM led to a reduction in net flow from 5.0 L/min (no AI) to 4.0 L/min (mild/moderate) to 1.8 L/min (severe). Increasing the speed to 6,400 RPM with severe AI and an uncoupled RV increased net flow by 45%, RF by 15% and reduced RV MVO2 by 1.1%. For the uncoupled RV with severe AI, blood pressure control alone led to a 22% increase in net flow, 4.2% reduction in RF, and 3.9% reduction in RV MVO2; pulmonary vasodilation alone led to a 18% increase in net flow, 7% reduction in RF, and 26% reduction in RV MVO2; whereas, combined BP control and pulmonary vasodilation led to a 113% increase in net flow, 20% reduction in RF and 31% reduction in RV MVO2. Compared to speed augmentation, blood pressure control consistently resulted in a reduction in WSS throughout the proximal regions of the arterial system.

**Conclusion:**

Speed augmentation to overcome AI in patients supported by CF-LVAD appears to augment flow but also increases RF and WSS in the aorta, and reduces RV MVO2. Aggressive blood pressure control and pulmonary vasodilation, particularly in those patients with an uncoupled RV can improve net flow with more advantageous effects on the RV and AI RF.

## Introduction

The development of *de novo* aortic insufficiency (AI) while on continuous flow left ventricular assist device (CF-LVAD) support is a common complication with up to 25% of patients developing mild to moderate AI within the first year after implantation (1–3). The severity of AI appears to be time-dependent with patients with longer durations of support developing more severe regurgitation. Nearly a third of patients will develop moderate or greater AI within 2–3 years of CF-LVAD implantation (1, 3). Over time, progressive AI may lead to LV chamber dilation followed by left-sided pressure elevation leading to pulmonary congestion. Eventually, secondary pulmonary hypertension leading to right-sided dysfunction may ensue.

Whereas, the prevalence of the disease is unmistaken, considerable controversy remains regarding the clinical significance and management of AI in patients supported with CF-LVADs. Cowger et al. performed serial echocardiograms on 166 patients following implantation with a CF-LVAD and found no difference in survival rates or rates of urgent transplantation following the development of moderate or worse AI (3). Despite the lack of a survival benefit, patients with moderate AI were more likely to develop mitral regurgitation, hemolysis, and worsening right ventricular (RV) dysfunction than patients without AI. In the subgroup of patients with pre-existing RV dysfunction prior to device implantation, patients who developed moderate or worse AI after CF-LVAD implantation had worse survival than those without important AI (3). Conversely, Jorde et al. followed 232 patients with CF-LVADs and found that 7 of 21 patients (33%) with moderate or greater AI developed symptoms of heart failure requiring urgent transplantation or aortic valve closure/repair (1). Forty percent of their cohort required an intervention within 3 months of developing symptomatic AI. Given the divergent conclusions from outcome studies examining the clinical consequences of AI in CF-LVAD patients, it is not surprising that there is a paucity of guidelines to help manage patients who develop important AI (4).

In practice, most clinicians increase LVAD speed in an attempt to overcome the regurgitant flow introduced by AI (5, 6). This increased speed increased the reverse transvalvular pressure gradient across the aortic valve which further results in earlier closure of the aortic valve, increases the regurgitant fraction and volume load on the LV and pulmonary circulation and consequently can lead to more pulmonary vascular remodeling and RV dysfunction over time. In severe cases of AI, surgical or transcatheter aortic valve replacement or closure can be performed although technical challenges related to lack or annulus calcification and progressive RV failure from abrupt changes in RV preload and afterload can ensue (7–9). It has been hypothesized that the ability of the right ventricle to handle volume challenges of AI and its management may be influenced by the RV coupling ratio. The RV coupling ratio defines the relationship of RV contractility, which can be estimated by the end-systolic elastance (Ees) of the RV, to the afterload or effective arterial elastance (Ea) of the pulmonary circulation. A coupled RV has sufficient contractile reserve to eject blood into the pulmonary circulation, whereas, an uncoupled RV struggles to eject blood, especially when challenged with high preload conditions.

Currently little is known about the functional impact of varying LVAD speed, blood pressure control and pulmonary vasodilators on patients with AI. The specific effects of different management strategies can often be hard to assess in clinical practice. Therefore, the development of strategies to help estimate the hemodynamic and clinical effects of these management strategies is of the utmost importance. We herein present a computational model of CF-LVAD patients with varying degrees of AI and RV-arterial coupling to determine, *in silico*, the effects of different management strategies, such as regulation of LVAD speed, blood pressure control, and administration of pulmonary vasodilator medications on cardiac function and shear stress distribution on the aortic wall.

## Materials and methods

The methodology is split into two phases. First, we built a closed-loop lumped-parameter model of the cardiovascular system to analyze global circulatory phenomena, with emphasis in the cardiac performance and its interaction with the LVAD. Second, we retrieved the hemodynamic conditions, specifically blood flow rates through the LVAD cannula and aortic root, and used them as boundary conditions to perform three-dimensional (3D) blood flow simulations using a patient-specific geometric model of the aorta and LVAD outflow cannula.

### Global circulation model

The closed-loop model accounts for the arterio-venous circulation, the four cardiac chambers with corresponding valves, the pulmonary circulation and the LVAD connecting the LV to the arterial system. Model parameters were selected to emulate the different physiological conditions of relevance for this study. We placed a HeartMate 3 (Abbott, Abbott Park, IL, United States) coupled with the LV in the closed-loop model. The model was developed and implemented in an in-house Python code. Full details of the model, including the model parameters used, have been previously reported (10). Ten cardiac cycles were simulated to ensure that all the variables in the model were in a periodic regime. Specifically, for the considerations of AI, the aortic valve was modeled taking into account the valve opening-closing dynamics (10). The pressure loss on the valve partially takes into account the high Reynolds number when the pressure-flow relation becomes non-linear (also known as turbulent loss). Evidently since there is no 3D modeling, the 0D representation is a simplified view of reality. The AI is modeled by modifying the parameters that control the minimum angle the valve can reach when it closes. Hypothetically, a perfect valve has a minimum angle of 0 degrees. In our model, we have modified this parameter so that the regurgitant fraction fell into the mild/severe classification (see definition below).

### Study cases and cardiovascular scenarios

Typical phenotypes of patients with LVAD and relevant clinical scenarios were defined by altering model parameters such as systemic and pulmonary resistance and compliance, cardiac elastance, and closing valve capabilities. To this aim, we used the computer simulations performed with the compartmental model. We hypothesized that the ability of the right ventricle to handle LVAD speed and volume changes may be dependent on the degree RV to pulmonary artery coupling. As study cases we defined four conditions combining the state of the right ventricle (RV) and the aortic insufficiency (AI):

1. Coupled RV and severe AI.
2. Uncoupled RV and severe AI.
3. Coupled RV and mild/moderate AI.
4. Uncoupled RV and mild/moderate AI.

The RV was deemed to be uncoupled when the ratio of RV Ees relative to the pulmonary effective arterial Ea was <0.7 and coupled when the ratio RV Ees/Ea was >0.7. Severe AI was defined as a regurgitant fraction (RF) of >50% and mild/moderate AI as a RF of <50%.

As cardiovascular scenarios we investigated the following protocols to counteract the pathophysiological conditions:

a) Baseline condition (HR 60 bpm, central MAP 80–90 mmHg, CO 5.0 L/min, mean pulmonary artery pressure 20–25 mmHg, LVAD operated at 5,500 RPM).
b) Left ventricular assist device speed augmentation (5,500 → 6,400 RPM).
c) Blood pressure (BP) control (target mean central aortic pressure 70–75 mmHg by reducing vascular resistance to 50–60% of its baseline value).
d) Pulmonary vasodilation (for the uncoupled scenarios, target systolic pulmonary pressure ∼25 mmHg by increasing pulmonary compliance by a factor of ∼10, and reducing vascular resistance to 80% of its baseline value).
e) Pulmonary vasodilation and BP control (for the uncoupled scenarios).

### Local circulation model

We simulated the 3D local blood flow in a patient-specific model of the aorta obtained through the segmentation of a computed tomography angiography dataset of a 50 years-old male patient who had a heartmate 3 (HM3, Abbott, Chicago, IL, United States) implanted at University of Chicago Hospital. Prior consent was obtained from patient, following the Declaration of Helsinki, and the imaging protocol as well as the use of the data was approved by the local ethics committee. We prescribed flow rate boundary conditions at the cannula inlet and at the aortic root as predicted by the global circulation model. At the five outlets (two subclavian arteries, two carotid arteries, and the descending aorta), resistance boundary conditions were prescribed to mimic the flow rate split occurring in the 0D model. Three cardiac cycles were simulated to ensure the solution becomes periodic, and the time-average wall shear stress (WSS) was computed for the last cardiac cycle in four different regions of the aortic model (outflow cannula, ascending aorta, aortic arch, and descending aorta). Details of the local circulation model have been previously reported (10). All simulations were conducted using an in-house simulation software (11). Simulations for the global circulation were run on a standard laptop, while simulations for the local circulation (3D) model were run in the Santos Dumont high performance facility (12).

### Model calibration

The proposed global-local model aims at characterizing the pathophysiological conditions encountered in a prototypical patient, to illustrate a proof-of-concept. The global circulation model was adjusted by performing a sensitivity analysis of the model predictions with respect to the main model parameters (perturbation in the range ± 40%). Specifically, these parameters were the systemic/pulmonary resistance/compliance, maximum/baseline cardiac elastances, and minimum valve opening angle. This sensitivity analysis led us to a baseline scenario, which was defined as the case that mimicked the main physiological conditions, as defined by physiological variables such as cardiac output, systemic/pulmonary blood pressure, end-systolic elastance, arterial elastance, and regurgitation fraction. From the baseline model, and exploiting the sensitivity analysis performed previously, we modified the model parameters to build virtual scenarios as those described in the previous section [state of the RV and the AI severity, see Table 14 in Blanco et al. (10)]. Concerning the local circulation model, although the 3D model of the aorta was built from patient-specific data, this 3D anatomical model was used here also as a typical anatomical model to investigate the sensitivity of the blood flow under the different conditions proposed above.

## Results

### Coupled right ventricular conditions

The introduction of mild/moderate or severe AI to the coupled RV and pulmonary artery at a speed of 5,500 RPM led to a reduction in net flow from 5.4 L/min (no AI) to 4.5 L/min (mild/moderate) and to 2.1 L/min (severe). RV Ees/Ea decreased from 1.01 (no AI) to 0.96 (mild/moderate) and to 0.76 (severe).

#### Effect of speed augmentation

**FIGURE 1:**
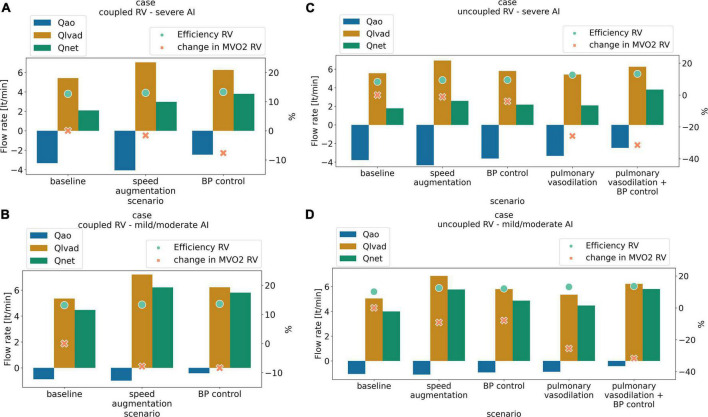
Antegrade flow (L/min) (orange), retrograde flow (L/min) (blue) net flow (L/min) (green) right ventricular cardiac efficiency (%) (dot light green), and change in right ventricular oxygen consumption (%) (RV MVO2, cross light orange) with medical management and LVAD speed augmentation in a scenario of **(A)** coupled right ventricular (RV) with severe aortic insufficiency (AI), **(B)** coupled RV with mild/moderate AI, **(C)** uncoupled RV with severe AI, **(D)** uncoupled RV with mild/moderate AI.

**FIGURE 2:**
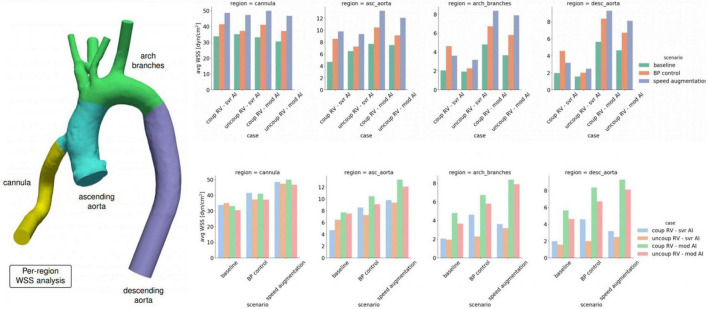
Average wall shear stress (WSS) by location within the left ventricular assist device (LVAD) outflow cannula or aorta stratified by degree of right ventricular (RV) coupling, aortic insufficiency (AI) severity and medical and device management.

**FIGURE 3:**
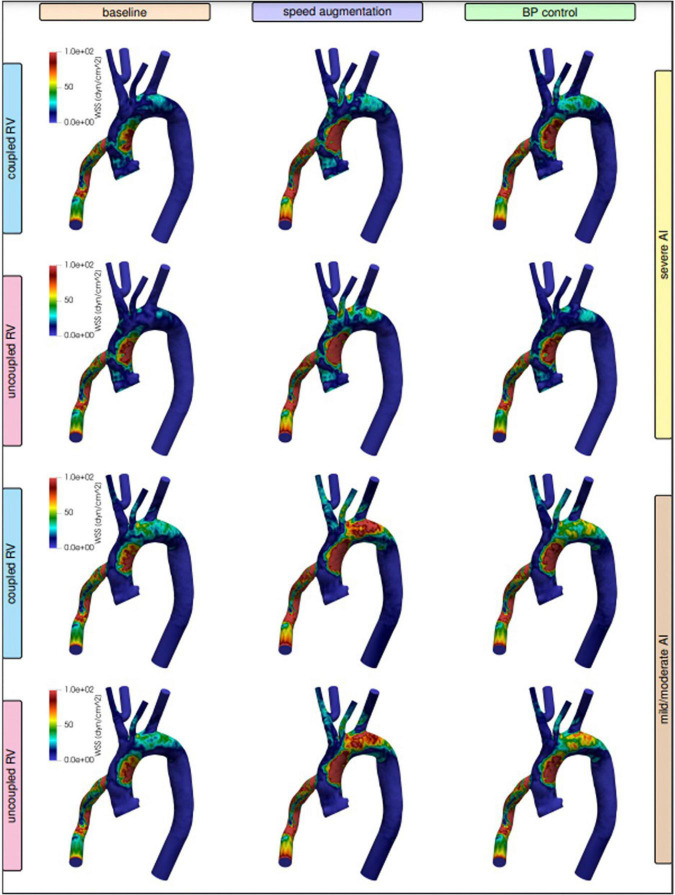
Visualized wall shear stress stratified by degree of right ventricular (RV) coupling and aortic insufficiency (AI) severity when comparing speed augmentation to blood pressure (BP) control.

Increasing LVAD speed to 6,400 RPM in the severe AI led to a 42% increase in net flow and a 16% increase in regurgitant flow (RF) with a nominal decrease of 1.6% in RV MVO2 (Table 1 and Figure 1A). Wall shear stress increased in the aortic tree and especially in the ascending aorta and was found to be two times higher than the baseline (average of 4.7 dyn/cm^2^ at baseline to 9.8 dyn/cm^2^ with speed augmentation) (Figures 2, 3). Speed augmentation in mild/moderate AI led to a 39% increase in net flow, 12% increase in RF and 8% reduction in RV MVO2 (Table 1 and Figure 1B) while the WSS was uniformly increased throughout the vascular tree with speed augmentation (Figures 2, 3).

**TABLE 1:** Intracardiac hemodynamics, energetics, left ventricular assist device (LVAD), and aortic flow with a coupled right ventricle with varying degrees of aortic insufficiency.

		Coupled RV–severe AI			Coupled RV–mild/moderate AI		
	Units	Baseline	Speed augmentation	BP control	Baseline	Speed augmentation	BP control
EesRV	mmHg/ml	0.364	0.360	0.356	0.364	0.351	0.354
EaRV	mmHg/ml	0.477	0.408	0.333	0.379	0.273	0.271
EesRV/EaRV	–	0.762	0.884	1.069	0.959	1.285	1.310
SWRV	mmHg.ml	851	860	827	862	805	816
MVO2RV	mlO2	0.0447	0.0440	0.0413	0.0435	0.0401	0.0398
PERV	mmHg.ml	726	684	589	659	555	532
PVARV	mmHg.ml	1,577	1,544	1,416	1,520	1,360	1,348
EffRV	–	0.127	0.130	0.133	0.132	0.134	0.136
Psa	mmHg	92	99	73	92	88	72
Psamax	mmHg	97	101	78	94	89	75
Psamin	mmHg	88	96	69	90	87	70
Ppa	mmHg	24	22	20	22	19	18
Ppamax	mmHg	28	28	26	27	25	25
Ppamin	mmHg	19	18	16	17	14	14
DPAoV	mmHg	57	67	43	58	63	44
Qao	l/min	−3.33	−4.07	−2.47	−0.87	−0.99	−0.42
Qlvad	l/min	5.42	7.05	6.26	5.35	7.20	6.22
Vback	ml	−59	−68	−50	−15	−16	−12
Net flow	l/min	2.10	2.97	3.80	4.47	6.21	5.80
Regurgitant fraction	−	61.3%	57.8%	39.4%	16.3%	13.7%	6.7%

#### Effect of blood pressure control

Blood pressure control in severe AI at 5,500 RPM led to an 81% increase in net flow with a 15% reduction RF and an 8% reduction in RV MVO2 while BP control with mild/moderate AI led to 30% increase in net flow, 18% decrease in RF and 8% reduction in RV MVO2 (Figures 1A,B). Blood pressure control in this setting was associated with a 15 and 13% reduction in WSS compared to speed augmentation in the outflow cannula and ascending aorta, respectively (Figures 2, 3).

#### Effect of simultaneous speed augmentation and blood pressure control

The combination of speed augmentation together blood pressure control in the scenario with severe AI and coupled RV led to a 48% increase in net flow compared to speed augmentation alone (4.40 vs. 2.97 L/min) and a 16% increase in net flow compared to BP control alone (4.40 vs. 3.80 L/min). Simultaneous speed augmentation and BP control modestly increased the regurgitant fraction of flow back across the aortic valve compared to BP control alone (42.8 vs. 39.4%) but was lower than isolated speed augmentation (57.8%). Overall, a strategy of simultaneous speed augmentation with blood pressure control was the most effective at improving net flow and only led to a modest increase in regurgitant flow when the RV is coupled.

#### Management strategy and regional wall shear stress

Local variations in WSS have been hypothesized to contribute to both the development and progression of AI. Here were observed regional variations in WSS by management strategy and degree of AI severity. With severe AI, WSS was lower in the cannula and ascending aorta but higher in the aortic arch and descending aorta using a blood pressure control strategy in favor of speed augmentation. Conversely, with mild or moderate AI, a blood pressure control strategy resulted in a uniform decreased in WSS throughout the entirety of the thoracic aorta when compared to a speed augmentation strategy (Figures 2, 3). Additional information on the pressure and velocity fields including the peak systolic to end-diastolic (S/D) velocity ratio and diastolic acceleration can be found in Supplementary Figure 1.

### Uncoupled right ventricular conditions

With an uncoupled RV, the introduction of mild/moderate or severe AI at a speed of 5,500 RPM led to a reduction in net flow from 5.0 L/min (no AI) to 4.0 L/min (mild/moderate) and to 1.8 L/min (severe). RV Ees/Ea decreased from 0.47 (no AI) to 0.38 (mild/moderate) and to 0.29 (severe).

#### Effect of speed augmentation

Increasing the speed to 6,400 RPM in severe AI scenario increased net flow by 45%, RF by 15% and decreased RV MVO2 by 1.1% (Table 2 and Figure 1C). WSS increased in all regions with the most notable augmentation occurring in the outflow cannula (35 dyn/cm^2^ at baseline vs. 47 dyn/cm^2^ with speed augmentation) (Figures 2, 3). With mild AI, speed augmentation led to a 44% increase in net flow, 4.5% increase in RF and 9% reduction in RV MVO2 (Table 2 and Figure 1D) with a consistent increase in WSS throughout.

**TABLE 2:** Intracardiac hemodynamics, energetics, left ventricular assist device (LVAD), and aortic flow with an uncoupled right ventricle with varying degrees of aortic insufficiency.

		Uncoupled RV–severe AI					Uncoupled RV–mild/moderate AI				
	Units	Baseline	Speed augmentation	BP control	Pulmonary vasodilation	Pulmonary vasodilation + BP control	Baseline	Speed augmentation	BP control	Pulmonary vasodilation	Pulmonary vasodilation + BP control
EesRV	mmHg/ml	0.340	0.331	0.339	0.364	0.356	0.327	0.315	0.326	0.364	0.354
EaRV	mmHg/ml	1.192	0.979	0.918	0.477	0.333	0.857	0.585	0.597	0.379	0.271
EesRV/EaRV	–	0.285	0.338	0.370	0.762	1.069	0.381	0.540	0.547	0.959	1.310
SWRV	mmHg.ml	754	842	822	851	827	891	994	977	862	816
MVO2RV	mlO2	0.0602	0.0595	0.0578	0.0447	0.0413	0.0584	0.0530	0.0538	0.0435	0.0398
PERV	mmHg.ml	1,564	1,445	1,384	726	589	1,345	988	1,038	659	532
PVARV	mmHg.ml	2,318	2,287	2,206	1,577	1,416	2,235	1,982	2,015	1,520	1,348
EffRV	−	0.083	0.094	0.095	0.127	0.133	0.101	0.125	0.121	0.132	0.136
Psa	mmHg	83	89	79	92	73	84	82	72	92	72
Psamax	mmHg	88	92	82	97	78	86	82	73	94	75
Psamin	mmHg	79	87	76	88	69	82	82	71	90	70
Ppa	mmHg	21	21	21	24	20	20	19	19	22	18
Ppamax	mmHg	41	39	39	28	26	38	34	34	27	25
Ppamin	mmHg	13	13	13	19	16	13	12	13	17	14
DPAoV	mmHg	57	72	53	57	43	69	75	55	58	44
Qao	l/min	−3.78	−4.34	−3.62	−3.33	−2.47	−1.05	−1.10	−0.93	−0.87	−0.42
Qlvad	l/min	5.56	6.92	5.80	5.42	6.26	5.05	6.86	5.80	5.35	6.22
Vback	ml	−63	−72	−60	−59	−50	−17	−18	−15	−15	−12
Net flow	l/min	1.78	2.59	2.19	2.10	3.80	4.00	5.77	4.87	4.47	5.80
Regurgitant fraction	–	67.9%	62.6%	62.4%	61.3%	39.4%	20.8%	16.0%	16.0%	16.3%	6.7%

#### Effect of blood pressure control

Blood pressure control alone in severe AI led to a 22% increase in net flow, 4.2% reduction in RF and 3.9% reduction in RV MVO2. Compared to speed augmentation, blood pressure control consistently resulted in a lower WSS in the entire aorta. More specifically, the WSS decreased in the aorta and especially in the outflow cannula by 21%, in the ascending aorta by 22%, in the aortic arch by 28%, and in the descending aorta by 19% (Figures 2, 3).

#### Effect of pulmonary vasodilation and combined pulmonary vasodilation and blood pressure control

Pulmonary vasodilation alone led to a 18% increase in net flow, 7% reduction in RF and 26% reduction in RV MVO2. A strategy that combined blood pressure control and pulmonary vasodilation led to a 113% increase in net flow, 20% reduction in RF and 31% reduction in RV MVO2 (Table 2 and Figure 1D). A strategy of simultaneous blood pressure control and pulmonary vasodilation was the most effective at improving net flow and reducing regurgitant fraction when the RV is uncoupled.

#### Management strategy and regional wall shear stress

Unlike with the coupled scenarios, there was no regional variation in vascular WSS when comparing a blood pressure control strategy vs. a speed augmentation strategy. Blood pressure control was associated with less WSS in the outflow cannula, ascending aorta, aortic arch, and descending aorta in both the uncoupled severe AI and uncoupled mild/moderate AI scenarios (Figures 2,3). While the S/D ratio was low for all scenarios, the diastolic acceleration was better able to discriminate mild/moderate from severe AI, especially when RV uncoupling was present (Supplementary Figure 1).

## Discussion

**FIGURE 4:**
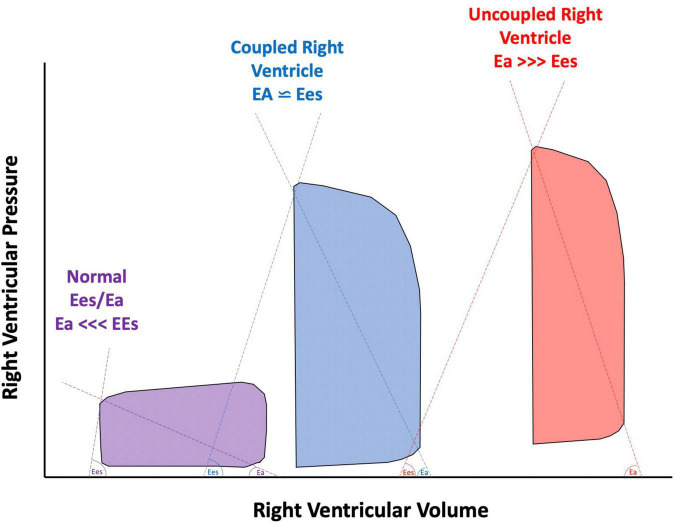
Pressure volume loops of a normal right ventricle (RV) compared to a compensated, coupled RV, and uncoupled RV. Ees, end systolic elastance; Ea, effective arterial elastance.

It is well-established that AI in patients supported with CF-LVAD has different pathophysiological implications than AI that develops in patients with unassisted hearts. With continuous unloading of the LV and return of blood to the ascending aorta, patients supported with CF-LVAD develop a constant trans-aortic pressure gradient which allows for continuous or pan-cyclical regurgitation of blood flow (13, 14). Therefore, the regurgitant blood volume is greater in AI in patients supported with CF-LVAD than in AI in native hearts where regurgitation only happens during diastole. This increased regurgitant volume increases LV filling pressures which over time can lead to both pre- and post-capillary pulmonary hypertension and increased myocardial workload for the RV (3, 15). With vascular remodeling, the effective arterial Ea increases and the ratio of the Ees of the RV to the effective arterial elastance (Ees/Ea) decreases. A coupled RV allows for a more efficient transfer of energy. Fortunately, the RV can maintain adequate efficiency up until an Ees/Ea of 0.7–0.8. Below this value, the RV becomes uncoupled and thus the mechanical efficiency of the RV reduces (Figure 4; 16–18). Receiver operating characteristic analysis has shown that an Ees/Ea of 0.7 has the greatest prognostic impact for a variety of clinical settings including chronic heart failure and pulmonary arterial hypertension (19).

Traditional management strategies in AI include speed augmentation in an attempt to overcome the regurgitant flow, and boost net forward flow into the aorta. With enhanced speed, an improvement in net flow can often be achieved but this strategy can cause progression in the aortic valve pathology by increasing the trans-aortic pressure gradient. From our simulations, speed augmentation led to an increase in average transvalvular pressure gradient that ranged from 8 to 26%, while blood pressure control led to reduction in mean pressure gradient values between 8 and 25%, with respect to the baseline case. A high trans-aortic pressure gradient promotes a better closure of aortic valve but could also induce aortic root dilation from increased circumferential stress (20). With more advanced disease, aortic valve replacement or occlusion can be considered although outcomes with these procedures have been variable and complicated by device migration, perivalvular leak and right ventricular dysfunction (6–8, 21, 22).

Here we showed that the optimal management strategy for AI differs by disease severity and the degree of RV coupling to the pulmonary circulation. Based on computational models of the circulation including the LVAD, the main findings of our work are as follows. (1) Speed augmentation increases net flow regardless of the degree of RV coupling and AI severity although it comes at the expense of increased WSS, increased regurgitant volume and transaortic pressure gradient promoting an AV closure. (2) In the setting of uncoupled RV, speed augmentation is less advantageous for the RV MVO2. (3) Tight blood pressure control either in isolation or combined with aggressive pulmonary vasodilation in those with uncoupled RV can achieve similar or greater augmentation in net flow with a reduction in RV MVO2, regurgitant volume and a reduction in the transvalvular gradient which will promote aortic valve opening. An aggressive management strategy was shown to be attainable in the Endurance Supplement trial where the HVAD arm had an average mean arterial pressure of less than 80 mmHg throughout the duration of the 24 months follow up (23).

**FIGURE 5:**
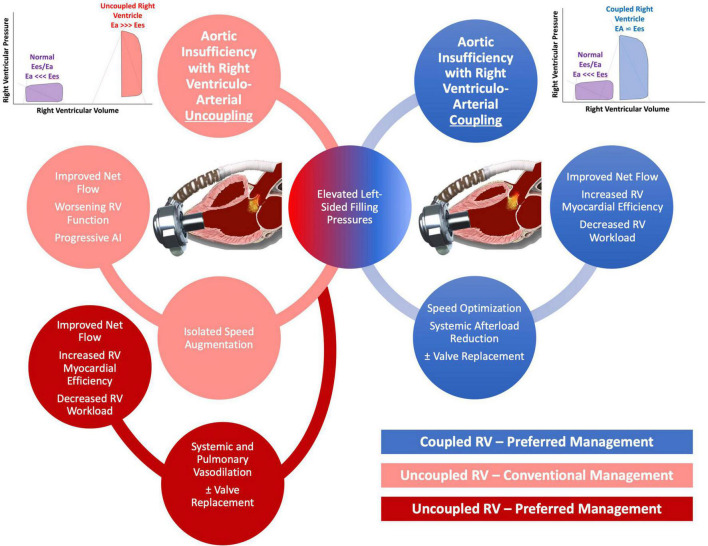
Schematic of proposed management of aortic insufficiency (AI) based on degree of right ventricular (RV) uncoupling.

Timely recognition of AI severity is of the utmost importance as management options are more abundant before fixed pre-capillary pulmonary hypertension and RV dysfunction ensues. Speed augmentation will increase preload to the RV but at the same time will reduce the elastance of the pulmonary circulation and thus the RV afterload. When AI is only mild/moderate or when the RV is coupled to the pulmonic circulation, our computer simulations showed that speed augmentation in addition to blood pressure control can improve net flow while having either neutral effects or even beneficial effects on RV energetics and workload. Conversely, when AI is more severe, particularly when the RV is uncoupled to the pulmonary circulation, speed augmentation has less advantageous effects on RV workload. Under these settings, the elastance of the pulmonary circulation is too high to accommodate the increased flow returning back to the RV which leads to an increase in RV pressure hence RV wall tension, and elevated myocardial workload. Unfortunately, these patients often already have a vulnerable RV and thus this management strategy can accelerate RV failure. In these setting, a strategy that aggressively reduces systemic and pulmonary pressures with a more judicious use of LVAD speed can augment net flow while at the same time reduce RV workload (Figure 5).

Recently, it has been recognized that traditional methods of quantifying AI may delay the diagnosis and underestimate disease severity. Instead, novel echocardiographic parameters obtained from the outflow cannula, the systolic to diastolic peak velocity ratio (S/D ratio) and the diastolic acceleration better prognosticate AI severity (24, 25). In our model, the diastolic acceleration was more predictive than the S/D ratio to discriminate mild/moderate from severe AI, especially when RV uncoupling was present. Better recognition and appreciation of the true AI severity in patients supported with CF-LVAD may allow for more timely non-invasive and invasive interventions that may improve clinical outcomes (26). In the absence of clinical symptoms, AI is often managed with diuresis, LVAD speed optimization and afterload reduction (4). Considerable uncertainty exists about the optimal strategy for adjusting the LVAD speed, especially when the AI becomes more severe. Reducing the speed of the LVAD to allow for at least intermittent aortic valve opening has been shown to reduce the rate of AI progression early in its course but the effect of aortic valve opening on the natural history of more severe AI is unknown (1, 27). Increasing the LVAD speed will unload the LV and reduce left-sided filling pressures but this will further increase the positive trans-aortic pressure gradient and the severity of regurgitation (1, 28). Increasing LVAD speeds during a hemodynamic ramp study can successfully overcome pulmonary capillary wedge pressure elevations in patients with AI to degrees comparable to those without AI (28). In the same study, AI severity worsened in nearly two thirds of patients who had AI at higher LVAD speeds although pulmonary capillary wedge pressure was successfully reduced in all but one patient despite the higher degree of AI. Increased LVAD speeds also led to normalization of low cardiac index in the majority of patients although cardiac index may remain low despite speed optimization if considerable RV dysfunction is present (28). Our work here suggests that when RV dysfunction is present or when a patient has an uncoupled RV, aggressive systemic blood pressure control and pulmonary vasodilation and to a lesser degree speed augmentation may be the preferred management strategy. The definitive management for AI in patients on LVAD support remains aortic valve replacement or closure or cardiac transplantation for appropriate candidates.

## Limitations

The computer simulations were performed using a model which was developed based on the physiologic and anatomical data for a single virtual patient with a HM3. Physiological data for this kind of patients was contrasted to model predictions to guide the selection of model parameters through a sensitivity analysis. Several variables including properties related to vessel and chamber elastance, and pulmonary and systemic resistance, had to be assumed based on previously published work to reflect prototypical patient phenotypes and treatment conditions. From the modeling perspective, the weak coupling between the 0D and the 3D models is a limitation. However, the pressure drop is mainly given by the cannula (see pressure field in the Supplementary Figure 1), and so the flow split in the major branches of the aorta will not be different from those prescribed from the 0D to the 3D model (which is actually determined by the downstream peripheral vasculature). Therefore, the only notorious discrepancy between the 3D and 0D models is the pressure pulse in the 3D, which is not realistic because of the rigid wall assumption (see systolic and diastolic pressure fields in the Supplementary Figure 1). In addition, since we employ the 3D model to estimate the regional WSS, and this depends on the flow rate, these results will not be greatly affected by the 3D–0D weak coupling. As said above, the velocity field is a direct consequence of the flow split among branches, being this the main determinant of the WSS regional distribution (see systolic and diastolic velocity fields in the Supplementary Figure 1). The pathogenesis of *de novo* AI in patients supported by LVADs can be heterogeneous and difficult to predict. Our model lacks a geometrically accurate representation of the aortic valve. The jet of RF can be eccentric or central and depends on the degree of commissural fusion, which could translate into different level of RF for the same geometrical area insufficiency. That being said, this assumption is commonly accepted in lumped-parameter model simulations. Moreover, our analysis focused on the effects of AI on the vasculature and RV and less on the impact on the LV. Lastly, our model assumed that RV Ees was relatively fixed with speed changes. With extremes of speed, the septal position can shift leftward which would impair RV Ees although in our experience, extreme shifts to this nature are rare in patients with AI as the regurgitant flow and concomitant elevated left-sided filling pressures tend to keep the septum in a more neutral position.

## Conclusion

Speed augmentation to overcome AI in patients supported by CF-LVAD will augment flow but at the expense of RV MVO2, RF, and WSS. Aggressive blood pressure control and pulmonary vasodilation, particularly in those patients with an uncoupled RV can improve net flow with more advantageous effects on the RV and aortic valve function.

## Data availability statement

The raw data supporting the conclusions of this article will be made available by the authors, without undue reservation.

## Ethics statement

The studies involving human participants were reviewed and approved by University of Chicago. The patients/participants provided their written informed consent to participate in this study.

## Author contributions

JG, PB, RT, and HG-G: study design. JG, PB, and CAB: study completion and data collection. JG, PB, and RT: clinical data analysis. JG, PB, CAB, CVB, PL, and HG-G: writing and editing. All authors contributed to the article and approved the submitted version.
