# Supplementary figures and images for: A computational study of aortic insufficiency in patients supported with continuous flow left ventricular assist devices: Is it time for a paradigm shift in management?
Source: Front Cardiovasc Med. 2022 Oct 20;9:933321. doi: 10.3389/fcvm.2022.933321 (PMC9631475; doi:10.3389/fcvm.2022.933321)

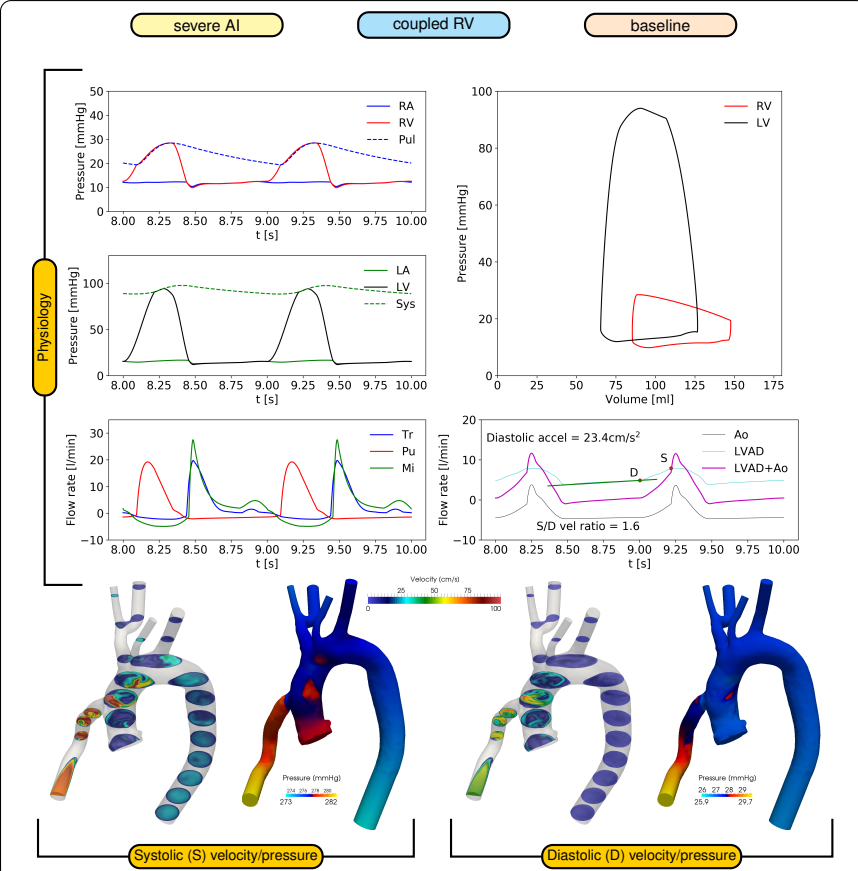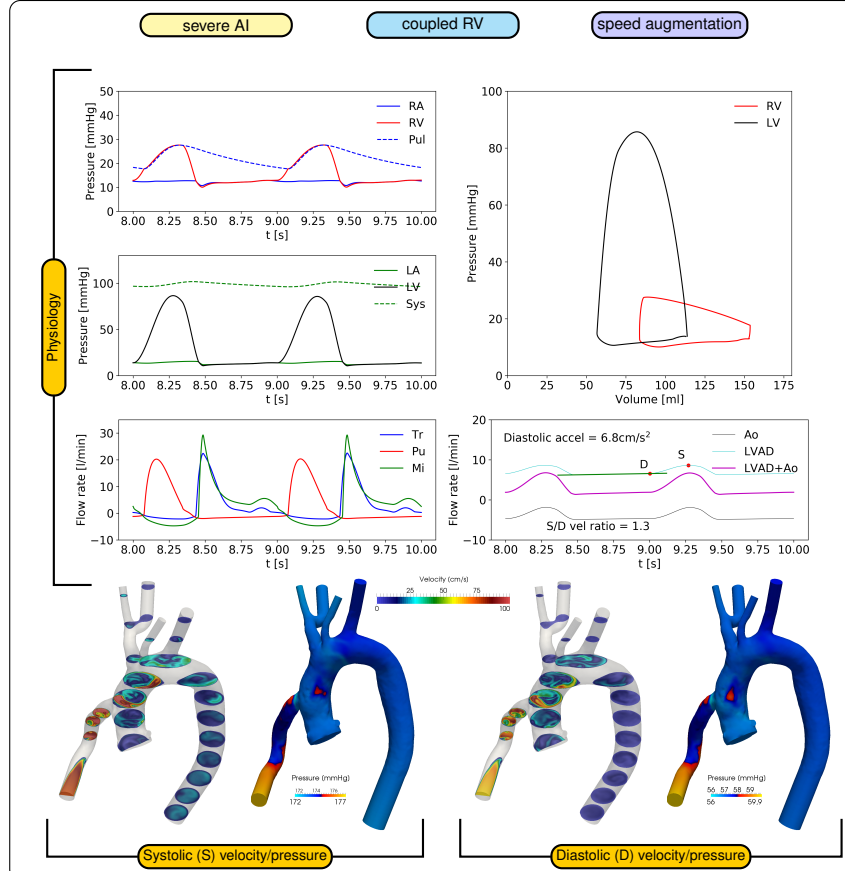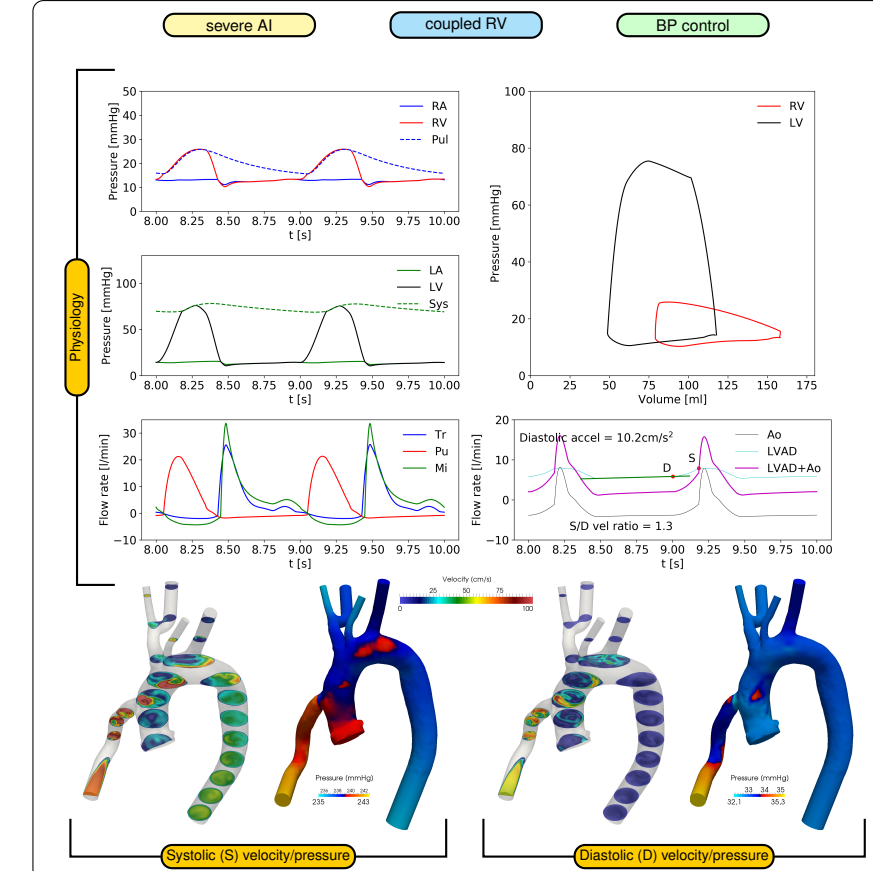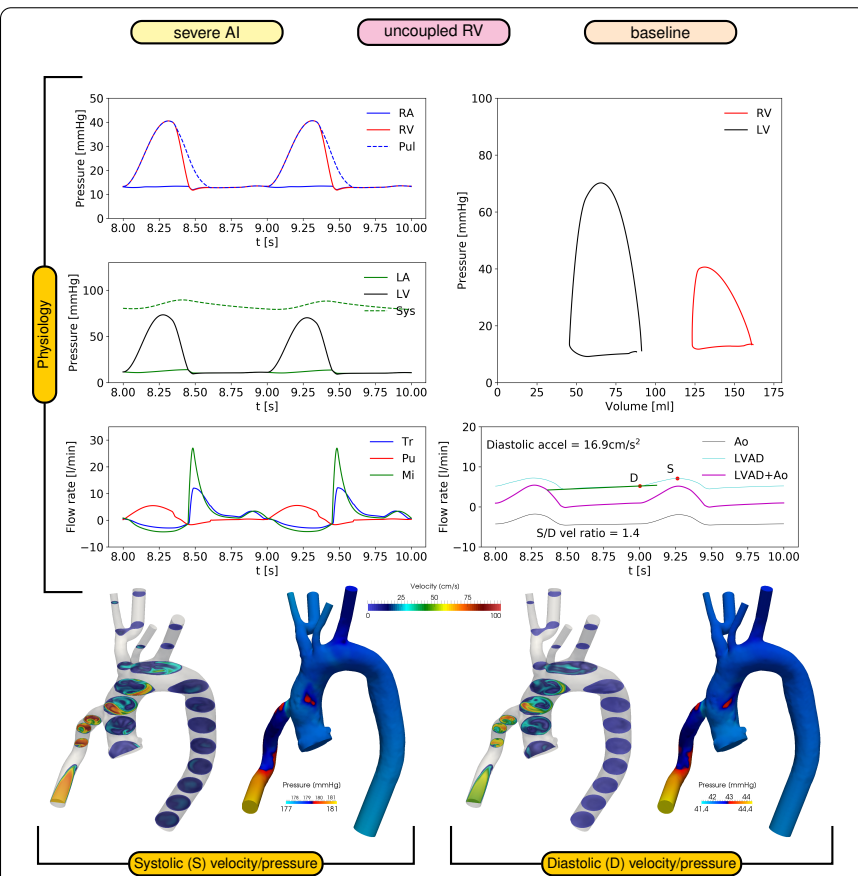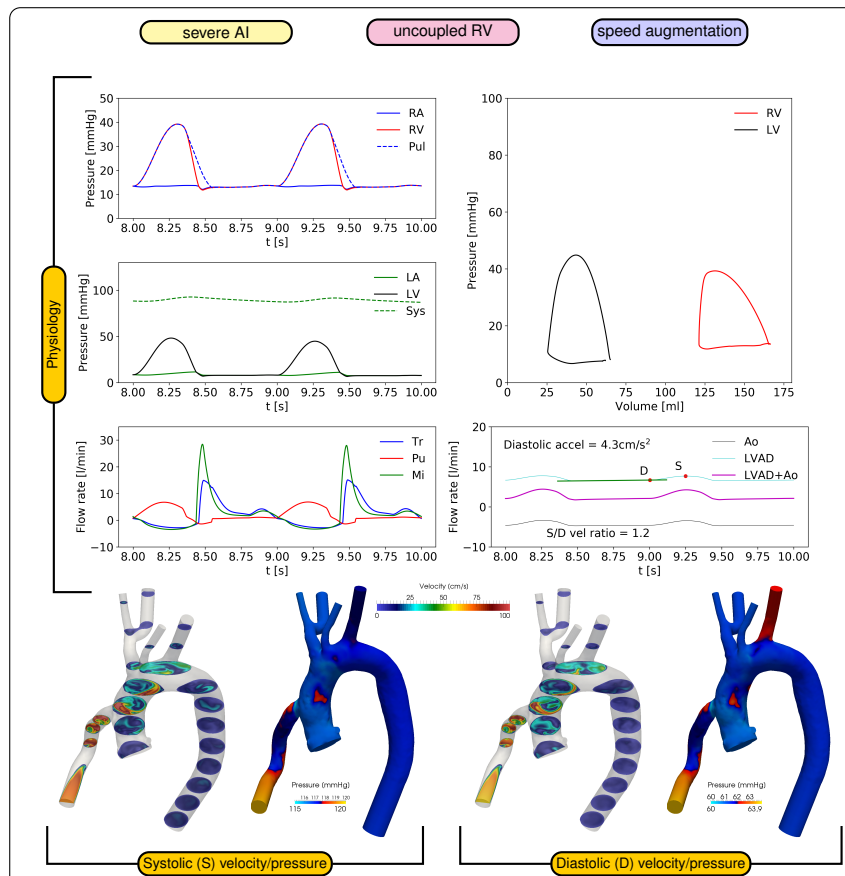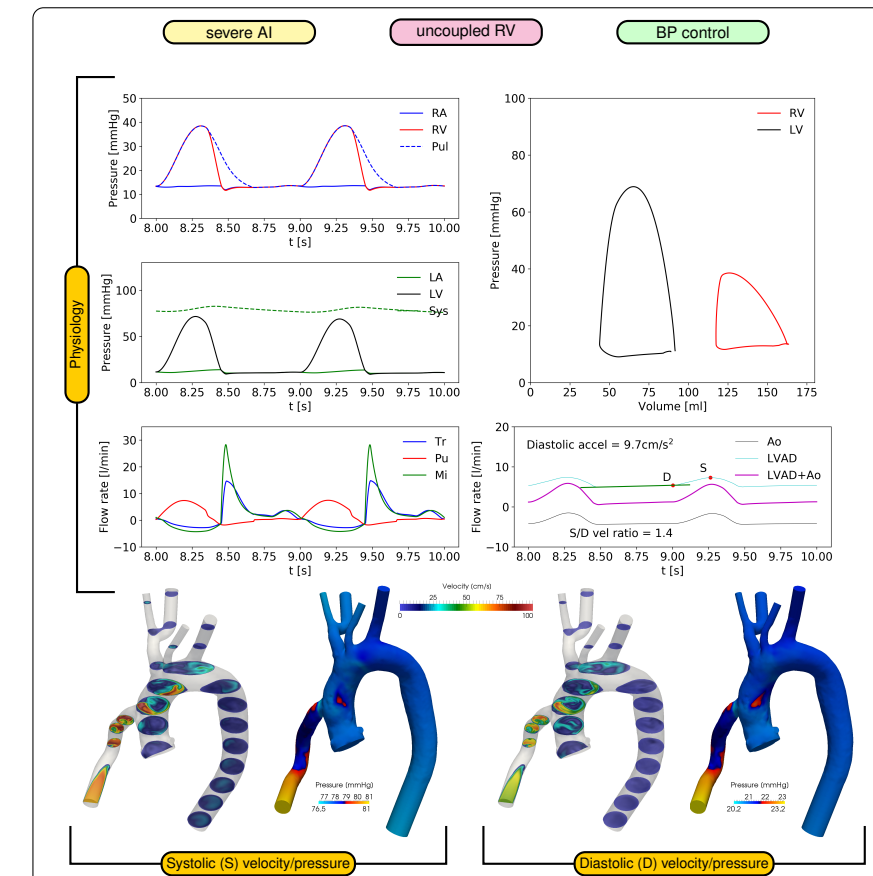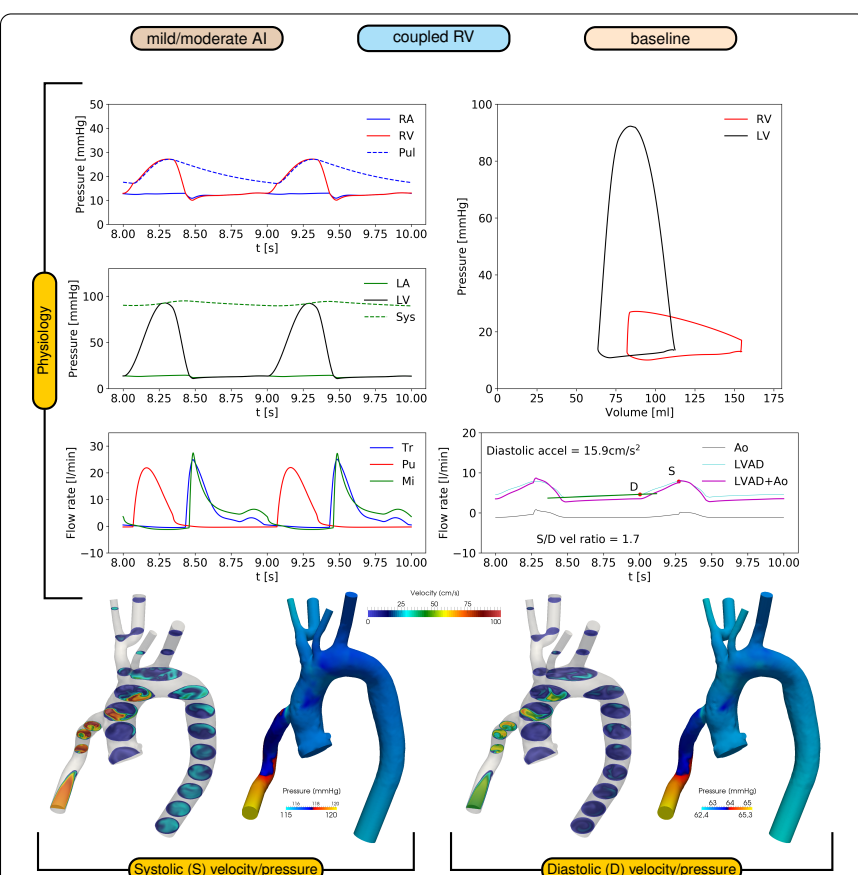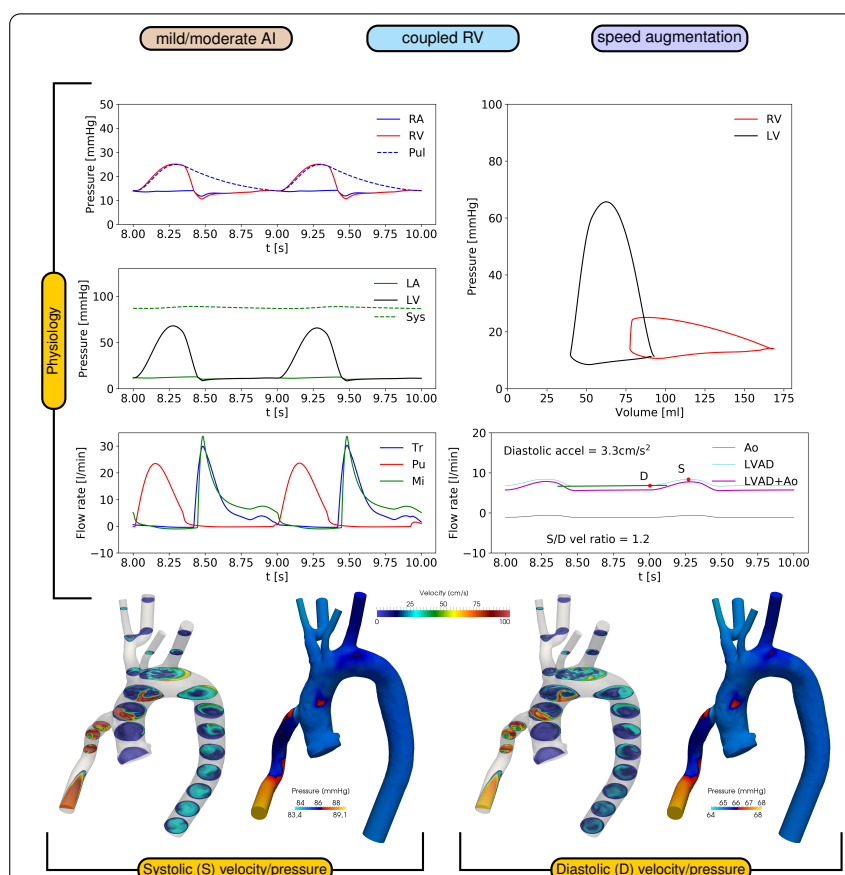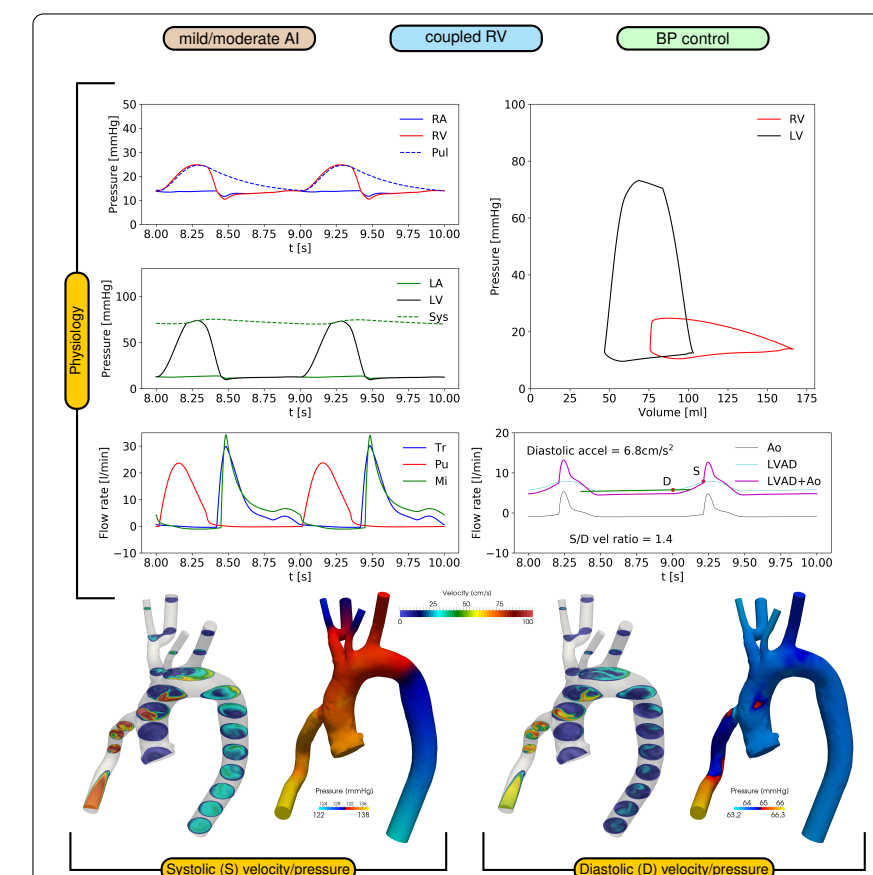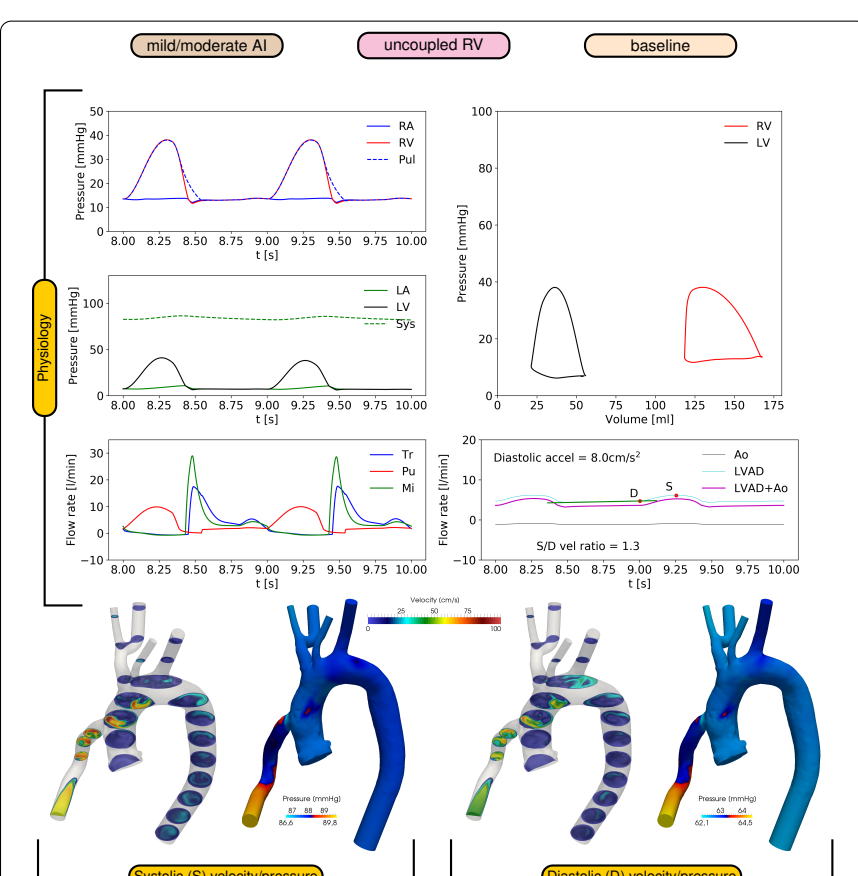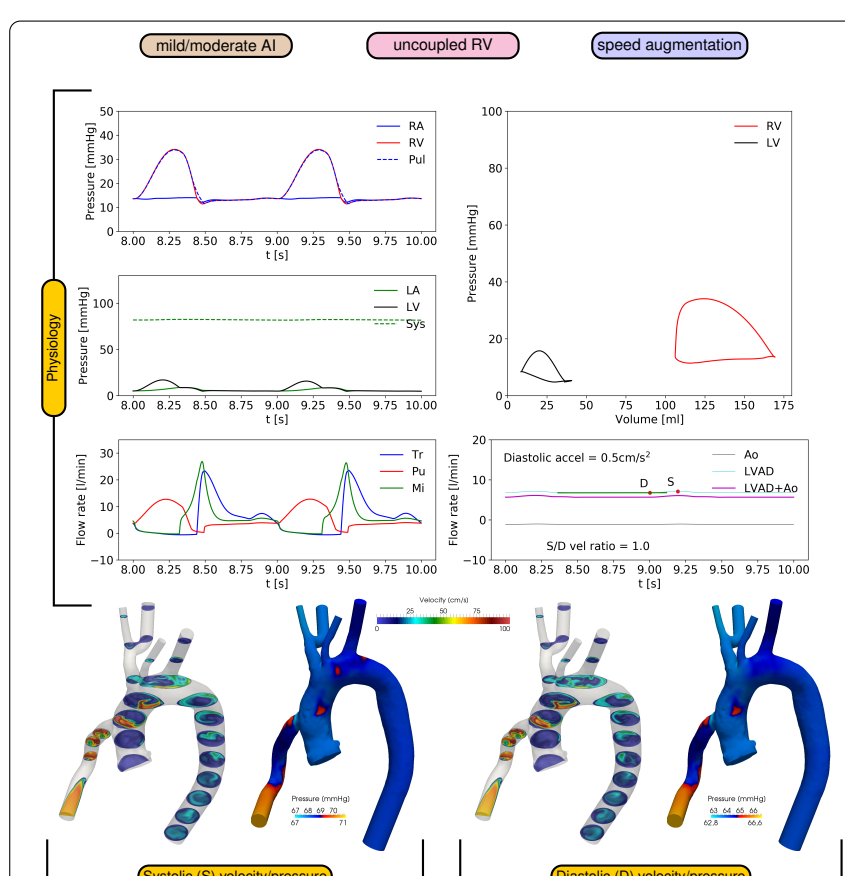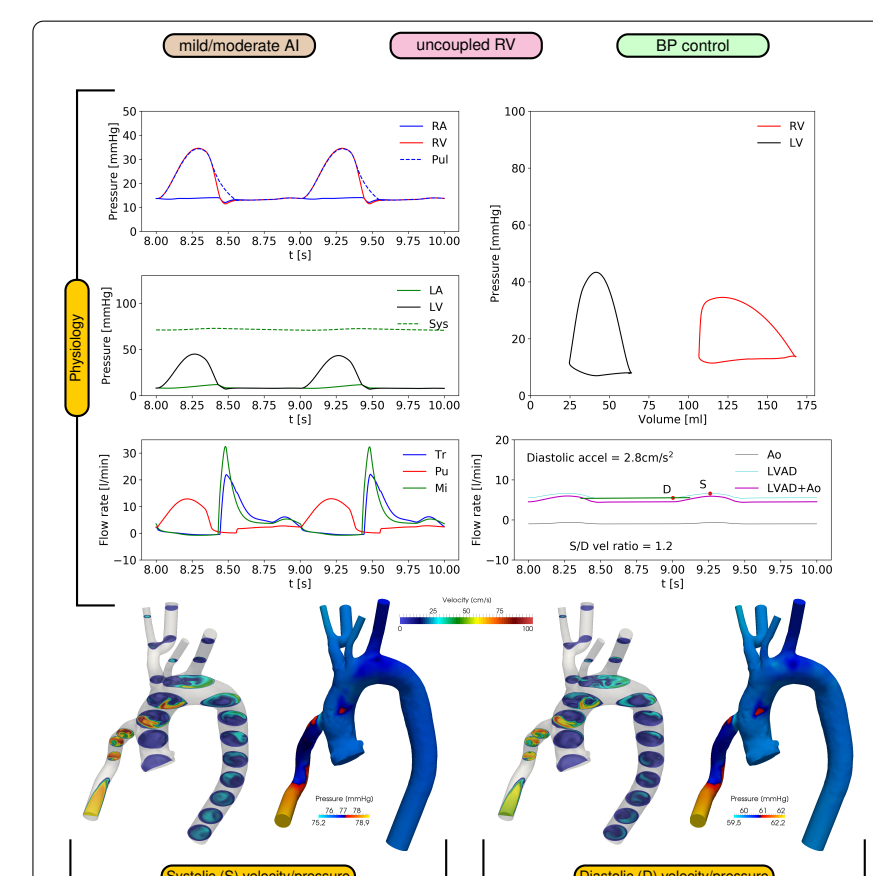

Supplement: Supplementary file 1 [file Data_Sheet_1.PDF]
